# In Silico Multitarget Profiling of Non-Selective Beta-Blockers Highlights Their Potential as Key Agents in Breast Cancer Adjuvant Therapy via ADRB2, ERBB2, and NPYR Receptors

**DOI:** 10.3390/cimb47100789

**Published:** 2025-09-23

**Authors:** Felipe Muñoz-González, José Correa-Basurto, Iván Ignacio-Mejia, Cindy Bandala

**Affiliations:** 1 Laboratorio de Diseño y Desarrollo de Nuevos Fármacos e Innovación Biotecnológica, SEPI-ESM, Instituto Politécnico Nacional, Mexico City 11340, Mexico; fmunozg2400@alumno.ipn.mx (F.M.-G.); jcorreab@ipn.mx (J.C.-B.); 2 Laboratorio de Neurociencia Traslacional, Escuela Superior de Medicina, Instituto Politécnico Nacional, Mexico City 11340, Mexico; ivanignacio402@gmail.com; 3 Laboratorio de Medicina Traslacional, Escuela Militar de Graduados de Sanidad, Centro de Investigación y Desarrollo del Ejército y Fuerza Aérea Mexicanos, Universidad del Ejército y Fuerza Aérea, Mexico City 11200, Mexico

**Keywords:** breast cancer, beta-blockers, drug repurposing

## Abstract

Breast cancer (BC) is associated with multiple molecular factors such as overexpression of the beta-2 adrenergic receptor (ADRB2) and the overproduction of its agonists (norepinephrine and epinephrine). The role of adrenergic signaling in BC highlights the therapeutic potential of non-selective beta-blockers (nsBBs) as inhibitors of well-established protumor signaling pathways related to ADRB2 and their possible affinity for other important protumoral receptors. Our aim was to identify how nsBBs currently prescribed may also interact with receptors other than ADRB2, which are related to the pathophysiology of BC, using bioinformatic intracellular pathway analysis (BIPA). Subsequently, the affinity of nsBBs for both ADRB2 and the targets identified by BIPA was evaluated. We found that, beyond ADRB2, both receptor tyrosine kinase 2 (ERBB2) and neuropeptide Y receptor (NPYR) are promising targets for nsBBs in the adjuvant treatment of BC, according to BIPA. Docking studies showed that the nsBB with the highest binding affinity (ΔG) was carvedilol (−10.5 kcal/mol), followed by propranolol (−8.5 kcal/mol). These in silico findings suggest previously unrecognized pharmacological mechanisms for nsBBs in the possible treatment for BC. Notably, differences in receptor affinity were observed among the nsBBs, with carvedilol exhibiting the strongest binding affinity values on ADRB2, ERBB2, and NPYR as biological targets against BC cells. These promising results require future experimental validation.

## 1. Introduction

Breast cancer (BC) is one of the most challenging health issues worldwide and is recognized as a multifaceted disease with significant impact on women’s health and public health systems globally. An important mechanism that contributes to the development of BC is chronic stress, supported by numerous studies, which indicate that the overproduction of catecholamines (Cas) linked to stress may influence cancer prognosis and mortality, affecting immune responses and cellular signaling pathways involved in carcinogenesis [1,2].

**Figure 1:**
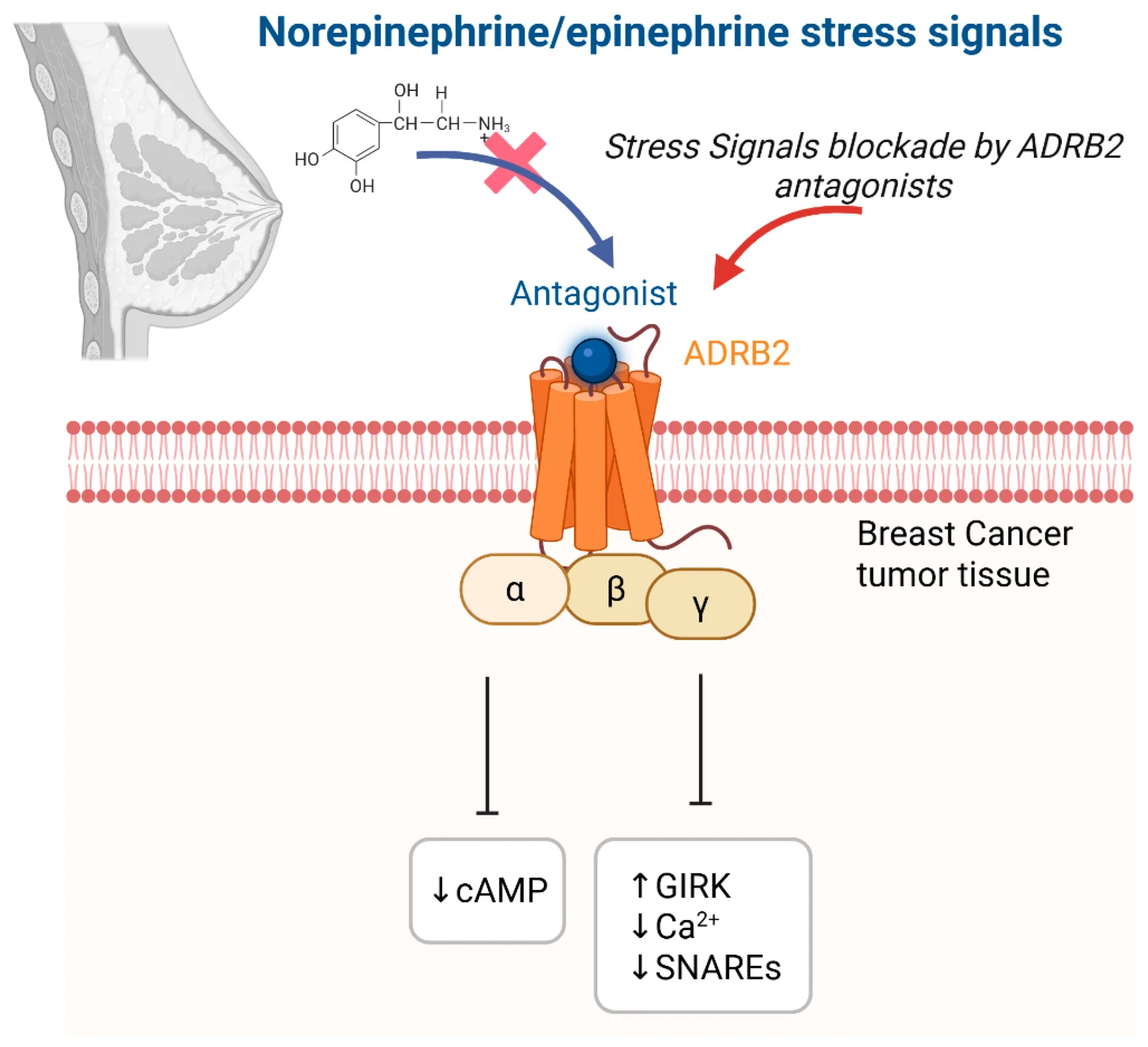
Antitumoral activity of nsBBs. Red cross represents the blockade of catecholamines stress sigaling by betablockers.

Chronic stress activates the beta-adrenergic receptors, primarily through Cas, such as epinephrine and norepinephrine, which bind to beta-2 adrenergic receptor (ADRB2). The Cas-ADRB2 complex activates a wide number of protumoral pathways [3]. The intracellular effects of Cas are mediated principally through non-bonded interactions with ADRB2, which results in metastasis in several cancer types, such as angiosarcoma, colorectal cancer, hemangioma, leukemia, lung, melanoma, nasopharyngeal, esophageal, ovarian, prostate, stomach and breast [4]. Experimental evidence indicates that excessive Cas can directly impact tumor cells and indirectly affect the tumor microenvironment to promote BC [5]. Chronic psychosocial stress has been found to activate the sympathetic nervous system (SNS) and the hypothalamic–pituitary–adrenal axis, leading to an aberrant release of Cas that accelerates cell proliferation, increased angiogenesis, immune evasion, and metabolic reprogramming, which fuel cancer growth and metastasis [6,7]. Non-selective beta-blockers (nsBBs) minimize the tumor-promoting effects associated with ADRB2 overexpression [7] (see Figure 1).

ADRB2 signaling influences nearly every tumor characteristic and has been linked with practically every stage of carcinogenesis and cancer progression [8,9].

Experimental studies have shown that nsBBs inhibit the effects of sympathetic nervous system activation, leading to reduced primary tumor growth and metastasis in mouse models of BC, and preventing tumor cell invasion [10]. In another study, treatment with nsBB reduced primary tumor growth and metastasis in a mouse model of BC [11].

Epidemiological and clinical studies have shown that the inhibition of ADRB2 signaling with nsBBs may decrease cancer progression and mortality in patients with BC [12,13,14]. A retrospective analysis revealed that women who were using nsBBs at the time of their BC diagnosis (n = 136) had lower breast cancer-specific mortality than those who were not (n = 3878) [15]. Hiller et al. [16] conducted a triple-blind, placebo-controlled clinical trial with 60 patients who were randomly assigned to receive an escalating dose of oral propranolol (the most studied nsBB in cancer) over one week. It is known that propranolol reduces intratumoral mesenchymal polarization and enhances immune cell infiltration in early-stage, surgically resectable BC. This effect is attributed to the inhibition of downstream pathways and the epithelial–mesenchymal transition (EMT) by propranolol, both in vitro and in vivo through the blockade of ADRB2 signaling [17,18]. We previously demonstrated in an observational study that propranolol reduced cancer metastasis at diagnosis by almost 50% compared with the group that did not receive it [19]; however, some reports are contradictory [10,20]. Another observed mechanism of nsBBs is their ability to reduce reactive oxygen species (ROS) production in MCF-10A cells, promoting chemopreventive activity [21]. These numerous pharmacological effects of nsBBs are explained by their low molecular weight and aromatic moieties, which allow them to reach various biological targets able to recognize molecules with these chemical properties [22]. Table 1 presents the IC50 values of propranolol and carvedilol related to their antitumor effects in BC s.

**Figure 2:**
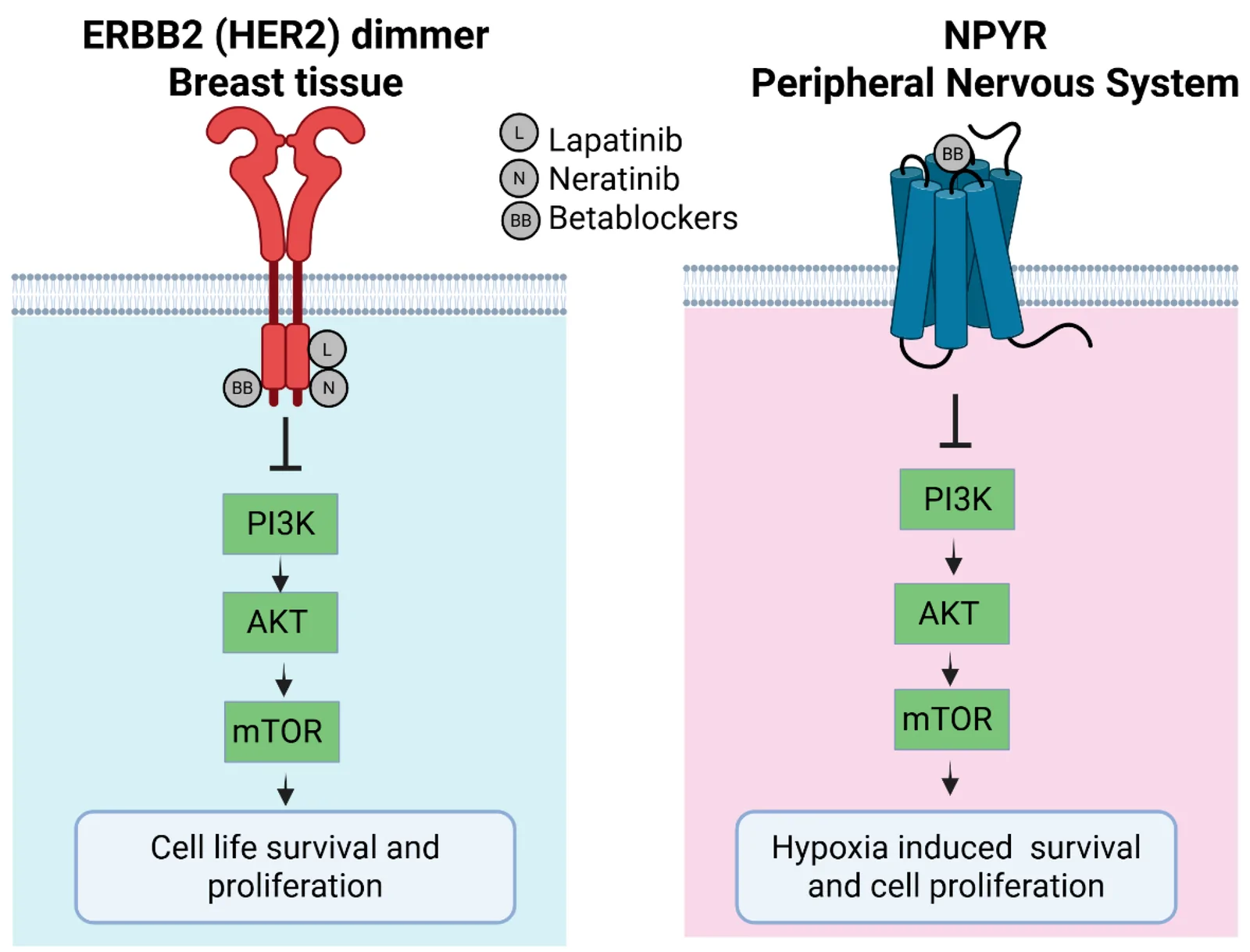
Proposed in silico interactions of non-selective beta-blockers with ERBB2 (HER2) and NPY receptors.

ERBB2 overexpression, observed in approximately 20–30% of BC cases, is strongly associated with uncontrolled cell growth, tumor aggressiveness, and poor prognosis [31]. Neratinib and lapatinib are currently approved small-molecule tyrosine kinase inhibitors (TKIs) that inhibit the catalytic activity of ERBB2 (Figure 2) by binding to its ATP-binding site, with lapatinib acting reversibly and neratinib irreversibly [32].

Physicochemical properties are crucial in drug design; one parameter related to permeability is LogP (log partition coefficient). LogP is one of the most critical parameters to consider in drug development to efficiently cross lipid-water barriers according to Lipinski’s rules. This is particularly important because the TK domain of ERBB2 is an intracytoplasmic protein. Cell permeability of drugs is critical for their inhibitory function. The LogP value is 4.72 for neratinib and 5.18 for lapatinib, both close to the desired values (LogP = 5). According to Lipinski’s rules, optimal membrane permeability is achieved with a LogP value between 1 and 3 [33]. Propranolol presented a LogP value of 3.03 and carvedilol 3.05 [34]. Therefore, nsBBs may represent a better option with fewer adverse effects [35], supported by solid preclinical and clinical evidence. Some drugs are known to be pharmacologically promiscuous, interacting with multiple biological targets. This is possible due to their lipophilic properties, small molecular weight, and aromatic moieties, which allow them to bind to various protein cavities, as is also observed with Cas.

On the other hand, neuropeptide Y (NPY) is a neurohormone found at high levels in human BC, where it signals through G-protein coupled receptors (GPCRs), primarily the NPY1 receptor (NPY1R) and NPY5 receptor (NPY5R), which are the most prominently expressed subtypes. While its abundance has been exploited for cancer imaging, interest in pharmacologically blocking NPY receptors to better understand their functional role in BC is growing [36]. It has been reported that 24% of BC cases express NPY2R, whereas 85% express NPY1R. Furthermore, important BC cell lines, such as MDA-MB-231 and MCF7, have elevated levels of NPY1R and NPY5R [35]. Given the dense sympathetic innervation and the abundant availability of NPY ligands in breast tissue, the NPY signaling pathway is likely to remain continuously active and to converge with ERBB2 downstream effectors (Figure 2). As a result, targeting NPY receptors with antagonists within the tumor microenvironment may represent a promising approach for BC treatment.

Propranolol has demonstrated promising anticancer effects, particularly in stress-sensitive tumors such as BC. Additional targeting of ERBB2 and NPYRs could enhance rational drug design and lead to the identification of novel modulators tailored for BC therapy, minimizing off-target effects while enhancing antitumor efficacy [36].

Therefore, the aim of this study was to explore, through a combination of bioinformatic intracellular pathway analysis (BIPA) and molecular docking simulations, the binding affinity of various nsBBs for three receptors associated with breast cancer: ADRB2, ERBB2, and NPYR. By identifying potentially relevant interaction profiles beyond the well-established propranolol–ADRB2 axis, this work seeks to provide preliminary in silico evidence that may support the development of future experimental studies focused on the multi-target potential of nsBBs in breast cancer therapy.

## 2. Materials and Methods

### 2.1. Molecular Docking

The following X-ray crystallographic structures from the Protein Data Bank (PDB) were used for molecular docking and structural analysis: 6PS5 (ADRB2), 3RCD (ERBB2), and 5ZBQ (NPY receptor).

### 2.2. Ligand and Protein Preparations

Ligands were retrieved from the PubChem database maintained by the U.S. National Library of Medicine [37]. The chemical structures of propranolol (CID_4946), (S)-Timolol (CID_33624), alprenolol (CID_2119), broranolol (CID_2475), carazolol (CID_71739), oxprenolol (CID_4631), carvedilol (CID_185395), pindolol (CID_688095), labetalol (CID_ 3869), nadolol (CID_39147), and sotalol (CID_5253) were downloaded as SDF 3D files and converted into .pdbqt format files using the Open Babel tool (https://openbabel.org/index.html, accessed on 6 July 2025), which was also used to perform energy minimization (structural optimization) and energy minimization with the universal force field (UFF), considering essential parameters such as atom types, hybridization states, and molecular connectivity. The PDBQT format was then obtained and prepared for molecular docking studies using AutoDock Tools 4.2.6 (https://vina.scripps.edu/, accessed on 6 July 2025).

### 2.3. Docking Simulations

The molecular docking analyses were performed using MGLTools and AutoDock Vina. AutoDock 4.2 and Vina were executed on a 64-bit Windows system with an AMD Ryzen 7 processor, 16 GB of RAM, and an AMD Radeon graphics card.

### 2.4. Target Preparation

The 3D PDB structures for all the receptors were prepared for docking via AutoDock Tools 4.2.6. All co-crystallized ligands and structural water molecules were removed. Polar hydrogen atoms and partial atomic charges were added to each receptor. Following this, the cleaned structures were saved in both *.pdb and *.pdbqt formats for subsequent docking analyses.

To perform docking studies of the selected ligands and the approved nsBBs, AutoDock Vina 1.1.2 was executed from the Linux terminal.

### 2.5. Docking Procedure (Vina)

The cleaned receptor structures were imported in .pdb format into AutoDock Tools 4.2.6. using the “Load Molecule” option. Then, the receptor structure was converted into the *.pdbqt macromolecule format compatible with AutoDock. Both ligands and targets, in the form of *.pdbqt files, were chosen for the docking process.

For molecular docking simulations, 3D grid boxes were defined as follows: ADRB2: (size_x = 70 Å, size_y = 70 Å, size_z = 70 Å; center_x = 0.0, center_y = 2.972, center_z = 55.000). ERBB2 (size_x = 78 Å, size_y = 56 Å, size_z = 56 Å, coordinates center_x = 5.39, center_y = 3.917, center_z = 26.189). NPY (size_x = 76 Å, size_y = 76 Å, size_z = 100 Å, coordinates center_x = −44.123, center_y = −22.327, center_z = 76.422). The AutoDock tools was used for this purpose, with exhaustiveness = 30 and number of modes = 10. Following the selection of molecules, active amino acid residues were designated to outline the cavity, using the “DockSiteScorer” option in ProteinPlus [38].

The grid box was appropriately aligned to encompass all active binding sites and essential residues for biological roles. The ligands and targets were subsequently subjected to docking studies to determine their binding affinities and identify binding cavities and active amino acid residues. The investigation of docking poses, as well as ligand and protein interactions, was conducted by importing the output files into PyMOL and ProteinPlus [38] in each protein–ligand complex, facilitating the identification of various types of non-bonded interactions.

### 2.6. Docking Validation

Re-docking was performed using AutoDock Vina and AutoDock4 to compare the ligand displacement calculated as RMSD values between ligand pose reported in the PDB crystal structure and the best pose (lowest-energy conformation) obtained by each program used. In the second step, the RMSD value obtained using Vina of each ligand (for propranolol, carvedilol, carazolol and labetalol) and these were compared with those obtained using AutoDock4. This procedure allows the accuracy of the docking to be evaluated by calculating the RMSD between the experimental conformation of the ligand and that generated by the docking algorithm. RMSD values less than 2.0 Å are considered indicative of an acceptable reproduction of the experimental binding mode [39].

### 2.7. Pathway Analysis and Combination

Protein–protein interactions (PPIs) are fundamental in constructing and interpreting PPI networks to elucidate the molecular mechanisms involved in oncogenesis and tumor progression, identify key proteins and complexes as potential therapeutic targets. The ability to integrate PPI data with other omics data can provide comprehensive biological information.

The STRING database (Available online: https://string-db.org, accessed on 18 July 2025) was used to construct an interaction network highlighting inflammatory processes associated with beta-adrenergic receptors and genes linked to BC. This is because the STRING database collects and integrates protein–protein interactions, both physical and functional associations [40]. The network shows the interaction between ERBB2 and ADRB2. ERBB2, also known as HER2, is a proto-oncogene that encodes a membrane receptor that regulates cell proliferation, motility, and apoptosis.

Bioinformatics tools and databases are essential for PPI network analysis and interpretation. A strong background in molecular biology, cell signaling, and oncology enhances the proficiency of cancer-related PPI research. In this context, integrated molecular docking could help assess the impact of modulating a pathway using agonists or antagonists.

The investigation involved the use of the advanced virtual screening platform STRING 11.0 [40] to explore the intricate network of interconnected proteins. The relevant genetic components were input into the STRING database, providing essential insights into multifaceted PPIs. Gene Ontology (GO) and Kyoto Encyclopedia of Genes and Genomes (KEGG) pathway annotations were performed using the ShinyGo platform [41]. The GO analysis aimed to examine the gene cluster within the network, enhancing the precision of the data prediction.

## 3. Results

### 3.1. Enrichment Analysis

**Figure 3:**
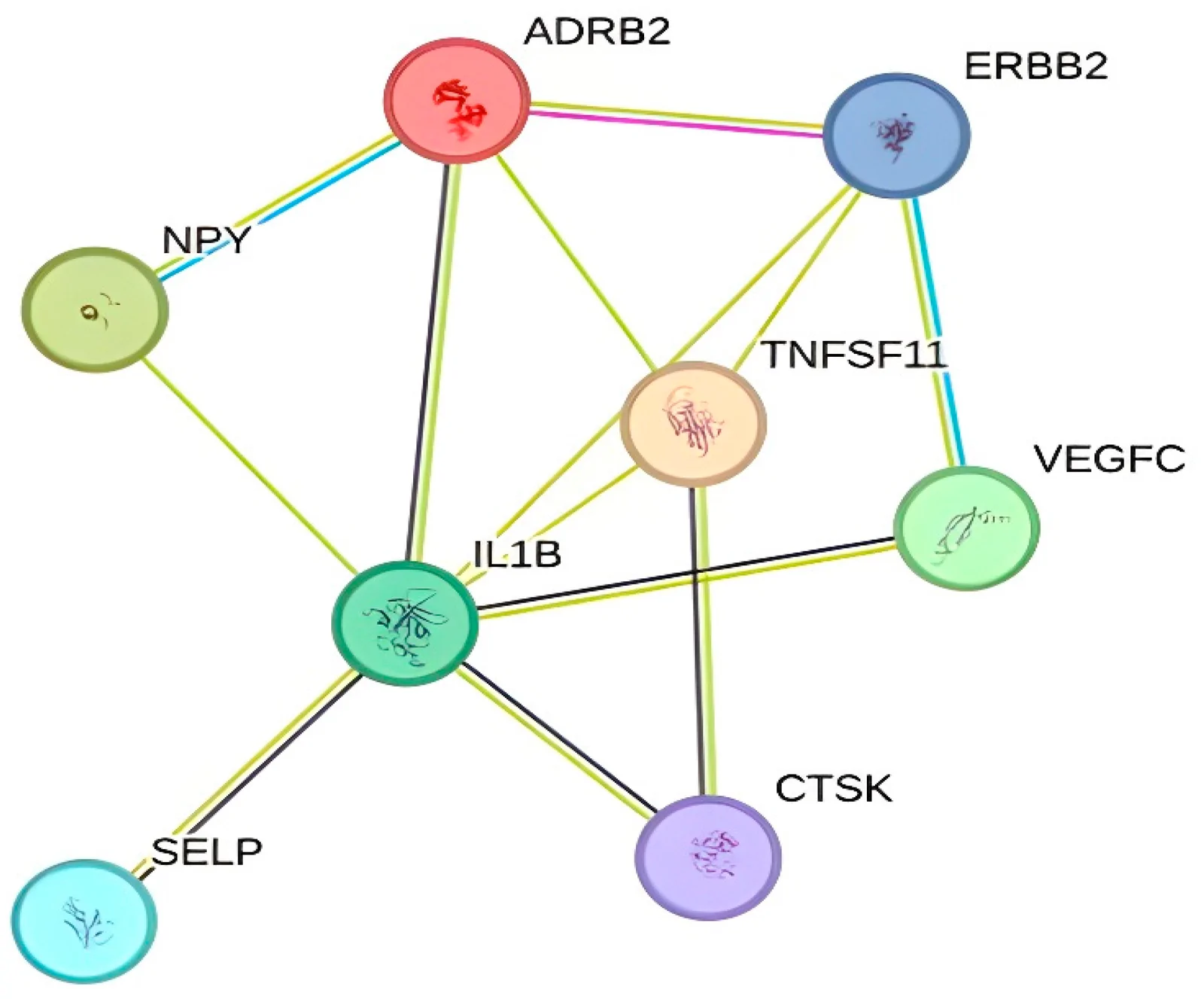
Protein–protein interaction network generated using STRING with ‘STRESS’ and ‘Breast Cancer’ as input terms. The red color edge for ADRB2 represents the first shell or central node. Colors of the circle for the second shell are randomly associated with all other proteins. Lines represent protein-protein associations, red lines: co-expression, green light lines: textmining, purple line: experimentally determined, blue line: from curated databases [40].

The complete protein–protein interaction (PPI) network (interactome), as a framework, provides a fresh perspective to address the challenges of traditional drug discovery and identify the subnetworks responsible for specific diseases and potential biological targets. We applied the STRING database [40] to construct a protein interaction network focusing on inflammatory pathways associated with ADRB2 and genes linked to BC such as IL1B and ERBB2. These proteins (Figure 3) are considered potential targets for molecular docking analysis due to their importance as first nodes in the network.

Previous research by Lourenço et al. [42] described stress-related proteins involved in BC development. The network revealed interactions among several proteins, including ERBB2, NPY, TNFSF11, and IL1-β, with ADRB2 as the first node in the network. The ERBB2 gene (also known as HER2) is a proto-oncogene encoding a membrane receptor involved in cell proliferation and survival. To better understand the biological processes involved in BC, we performed a Gene Ontology analysis, summarized in Table 2.

IL-1β was associated with ADRB2 in the interaction network, supporting recent findings on the role of hypoxia in BC progression and the identification of additional therapeutic targets. This occurs through IL-1β/IL-1R1 signaling, which is activated under hypoxic conditions and drives aggressive behavior in triple-negative BC (TNBC) and cancer-associated fibroblasts [43]. KEGG pathway analysis (Table 3) revealed that the regulation of lipolysis in adipocytes is a pathway implicated in BC. Adipocytes, which are the main components of breast tissue, are also associated with cancer, constituting key elements of the tumor microenvironment in BC [44,45]. Additionally, osteoclast differentiation is upregulated in BC, which facilitates bone metastasis, disrupting the normal bone remodeling process and leading to bone loss. This process is mediated by osteoclasts, which are responsible for bone resorption. As osteoclasts break down bone, they release factors that further support the growth of tumors in the skeletal system. Tumor cells can induce lipolysis in adipocytes, releasing free fatty acids (FAs), which are taken up by tumor cells, enhancing invasiveness and metastatic potential [46]. Survival and apoptosis-related mechanisms in BC may be modulated by ADRB2 antagonists. Myocardial ERBB2 and ADRB signaling are linked in a feedback loop, where ADRB activation upregulates ERBB2 expression and activity, forming a self-reinforcing feedback loop [46]. In the same network (Figure 3), IL-1β interacts with ADRB2; hence, recent findings highlight mechanisms through which the hypoxic tumor microenvironment may contribute to BC progression and suggest additional therapeutic targets. This occurs via hypoxia-induced IL-1β/IL-1R1 activation [47]. In murine models, IL-1β deficiency or its blockade with specific antibodies resulted in regression of BC. This effect is attributed to reduced macrophage-mediated immunosuppression and enhanced antitumor immunity, facilitated by activated dendritic cells and cytotoxic CD8+ lymphocytes [48].

### 3.2. Molecular Docking Simulations

To evaluate the binding affinity of tested nsBBs to proteins involved in BC-associated pathways and the protein interaction network; molecular docking was performed using the main protein targets: ADRB2, NPYR, and ERBB2. The rationale for selecting these proteins lies in their significant involvement in PPIs, compound–target network construction via STRING, KEGG analysis [49], and Cytoscape 3.10.3 [50]. In addition, these proteins possess ligand-binding pockets capable of accommodating small, aromatic molecules (PDBs: ADRB2, 6PS5; ERBB2, 3RCD; NPYR, 5ZBQ) able to accept Cas. Hence, this exploration consisted of an assessment of the ligands’ binding conformations and predicted affinities to evaluate their possible impact on the protein network. We compiled the molecular docking results between the 11 test ligands and the ADRB2, ERBB2, and NPYR receptors in Table 4, Table 5, and Table 6, respectively.

**Figure 4:**
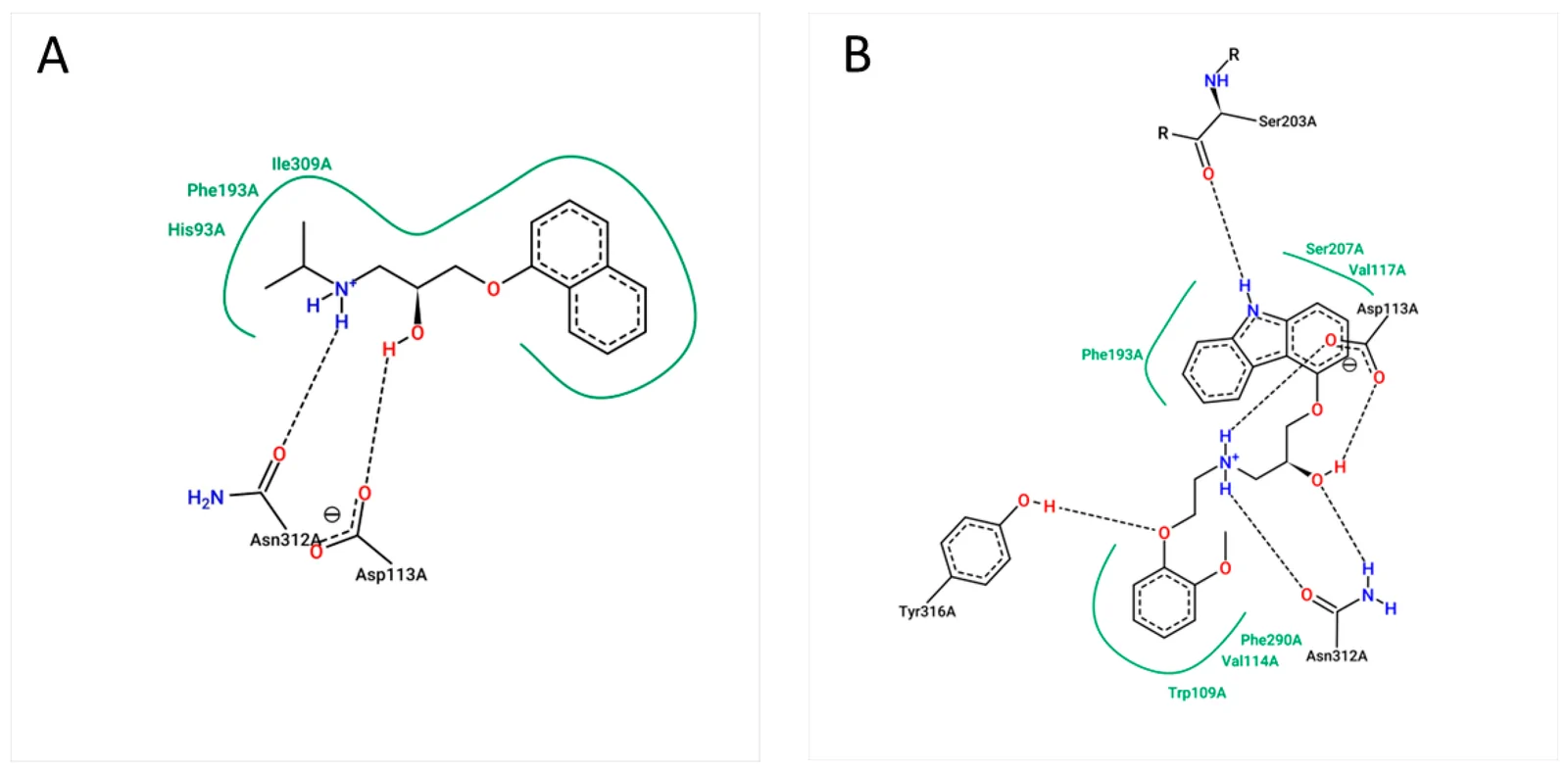
Docking interactions of the main approved BBs on ADRB2 and Cas: binding residues for each ligand are shown. (**A**) Propanolol, (**B**) Carvedilol. Dotted lines represent hydrogen bonds, green lines indicate hydrophobic interactions.

**Figure 5:**
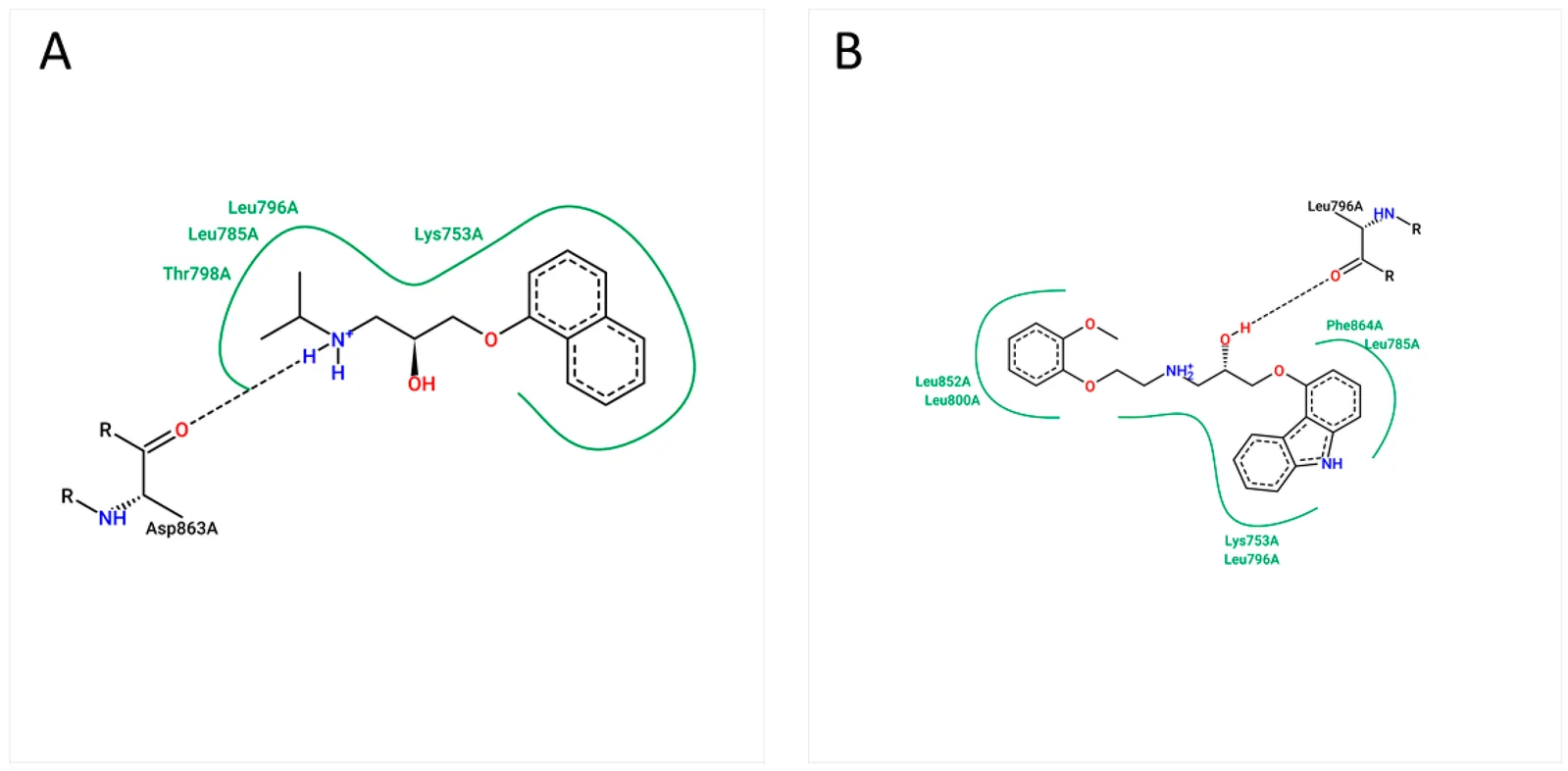
Docking interactions of approved BBs on ERBB2 and Cas: binding residues and interaction types. (**A**) Propanolol, (**B**) Carvedilol. Dotted lines represent hydrogen bonds, green lines indicate hydrophobic interactions.

**Figure 6:**
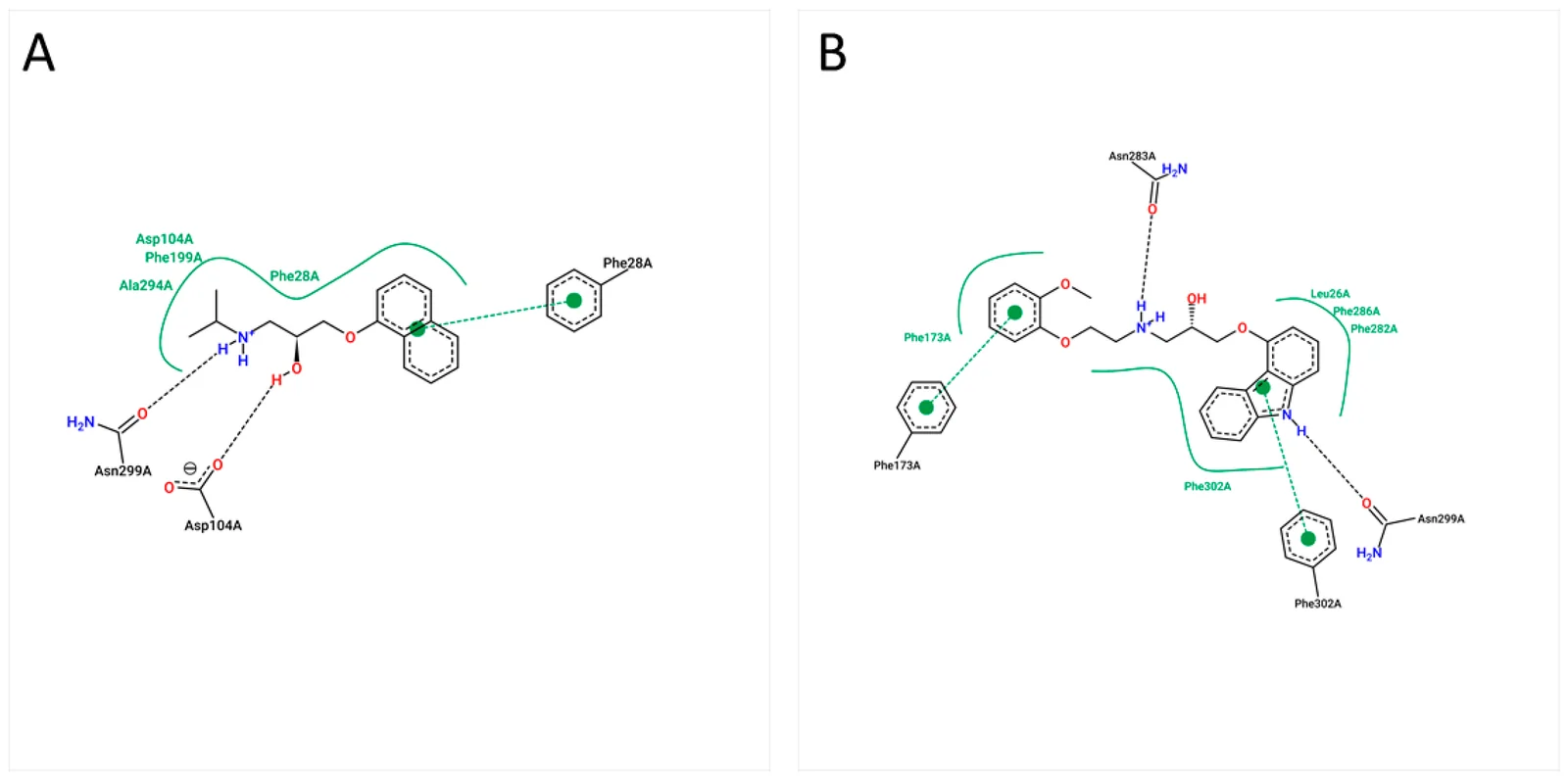
Docking results for BBs on NPYR and Cas: binding residues and interaction types. (**A**) Propanolol, (**B**) Carvedilol. Dotted lines represent hydrogen bonds, green lines indicate hydrophobic interactions, and green dots denote aromatic interactions.

The binding energy calculated by the VINA program is included, along with hydrogen bond interactions, nonpolar (hydrophobic) interactions, and π-type (aromatic) interactions (Figure 4, Figure 5 and Figure 6), all of which are calculated using the ProteinPlus web server [38]. A comprehensive examination of signaling pathways and pathological conditions associated with specific genes is essential for exploring the structure-based design of molecular compounds [51]. Detailed insights into the docking scores, ligand conformations, and their interactions with ADRB2 are illustrated in Figure 4, Figure 5 and Figure 6. Additionally, propranolol was shown in this study to interact with the main proteins of the interactome constructed in STRING with the terms “Breast Cancer” and “ADRB2”.

### 3.3. Results of DockingValidation

The docking protocol was validated using a redocking approach, in which the original ligand from the crystallized protein structure is removed and then re-docked into the same binding site using the tested protocols. The accuracy of redocking was assessed by comparing the predicted pose to the experimental X-ray crystallographic structure and calculating the root mean square deviation (RMSD) (Table 7).

This procedure allows the accuracy of the docking to be evaluated by calculating the root mean square deviation (RMSD) between the experimental conformation of the ligand and that generated by the docking algorithm. RMSD values between 2.0 and 3.0 Å are considered indicative of an acceptable reproduction of the experimental binding mode [39]. To perform this structural analysis, the PyMOL 2.3.0 software was used, the receptor and ligand were prepared independently; the original active site was defined; the docking protocol was executed, and finally, the RMSD between the predicted lowest energy pose from docking simulations and the original structure of the co-crystallized ligand was calculated. We also included a comparison between the best pose obtained by Vina (reference program) and those generated by AutoDock4 (Table 8). All data and images related to redocking validation was included as Supplementary Materials (Suplement 1. RMSD Images).

## 4. Discussion

We studied the pharmacological potential of 11 nsBBs for possible BC treatment. First, we performed a BIPA, identifying ERBB2 and NPYR as potential targets of nsBBs with similar affinity to ADRB2. Structural analyses revealed that these three biological targets can recognize Cas and nsBBs due to their cavities being occupied by small and aromatic molecules, as shown in their 3D structures reported in the PDB. Docking studies revealed that the main ADRB2 ligands (propranolol, carvedilol, carazolol and labetalol) have strong hydrophobic and aromatic interactions as well as hydrogen bonds that stabilize protein–ligand complexes, which have experimental support [52]. The compounds with the strongest binding energies for ADRB2 are carvedilol, carazolol and labetalol (−10.5 kcal/mol, −9.5 kcal/mol and −9.2 kcal/mol, respectively); even propranolol, with a weaker affinity (−8.5 kcal/mol), could still be considered a strong ligand because it interacts with ADRB2 through two hydrogen bonds with Asp113 and Asn312, a site where Cas are recognized [53]. Sloan et al. have reported that sympathetic nervous system (SNS) activation promotes BC metastasis through β-adrenergic signaling, which recruits macrophages into the primary tumor and induces a pro-metastatic gene expression signature. The study also revealed that propranolol treatment blocked stress-enhanced metastasis in animals subjected to chronic restraint stress [54]. Carvedilol shows the greatest number of interactions through hydrogen bonds with Asp113, Asn312, Tyr316, and Ser203. This is because carvedilol is a third-generation compound designed to maximize affinity for the active site, because it possesses a markedly different core structure compared with first-generation beta-blockers such as propranolol and atenolol, which increases its affinity for β2 agonists. Carazolol interacts with serotonin receptors, particularly the 5-HT1A receptor. There is preclinical evidence suggesting that signaling through certain serotonin receptor subtypes may be involved in processes that promote oncogenesis, such as angiogenesis, proliferation, and cell migration. For example, studies have shown that the activation of receptors such as 5-HT2A and 5-HT2B can promote the formation of new blood vessels and stimulate tumor growth in certain types of cancer, such as breast, colon, or melanoma [55]. Consequently, some researchers have explored the use of antagonists of these receptors. For example, ketanserin, a 5-HT2A receptor antagonist, has shown antiangiogenic and antiproliferative properties in vitro and in vivo, suggesting potential as an anticancer therapy [56]. For this reason, carazolol could be a promising candidate for evaluation as an anticancer drug. In contrast, there is preclinical evidence that labetalol acts as a growth inhibitor in neuroblastoma cells with potency comparable to propranolol [57].

The ERBB2 gene, encodes a protein involved in cell growth and programmed cell death. ERBB2 is a receptor tyrosine kinase (RTK) that contributes to normal cellular processes. However, in certain cancers, including breast, ovarian, bladder, pancreatic, stomach, and esophageal cancers, overexpression of the ERBB2 protein occurs [58]. Our docking studies revealed moderate interactions of propranolol (−6.9 kcal/mol) with the ERBB2 protein; in contrast, carvedilol and labetalol exhibited stronger interactions (−9.1 and −8.8 kcal/mol, respectively), and these values represent the strongest affinities among all tested ligands. This result is relevant because ERBB2 inhibitors, such as lapatinib (Tykerb), a TKI that blocks growth signals in HER2-positive cancer cells, are clinically used in combination with other treatments for advanced BC. Neratinib (Nerlynx) [59], another TKI that targets HER2 and EGFR, is used in the adjuvant treatment of ERBB2-positive BC, and tucatinib. TKI that selectively binds to ERBB2 and has shown efficacy in combination with other agents in ERBB2-positive metastatic BC [60].

Finally, molecular docking simulations were applied to the NPY1 receptor. In this context, carvedilol showed a strong affinity (−9.2 kcal/mol) for the NPY1 receptor, which was the highest value. Additionally, nadolol, labetalol, and carazolol exhibited strong affinities (−9.0, −8.5, and −8.4 kcal/mol, respectively). These results are similar to those obtained for ADRB2 docking, which is plausible, as both are members of the GPCR family. This result is remarkable because BC can display various peptide receptors, such as somatostatin, vasoactive intestinal peptide (VIP), gastrin-releasing peptide (GRP), and NPY1 receptors, and recently, studies have indicated that NPY1 receptors are also widely expressed in BC cells studied in vitro [61].

For the re-docking analysis, RMSD values were calculated by comparing the best pose obtained with Vina against the crystallized ligand poses in the PDB receptor structures ADRB2, ERBB2, and NPYR; the RMSD values for Vina were <2.0. Additionally, redocking with AutoDock4 yielded RMSD values between 2 and 3. Both Vina and Autodock were suitable for downstream docking analysis. Subsequently, docking analysis was performed for the test ligands propranolol, carvedilol, carazolol, and labetalol. The results of redocking showed that Vina and AutoDock4 were consistent in calculating RMSD values.

Our results, as previously explained, are promising for the adjuvant treatment of BC. Although some nsBBs, such as propranolol, have been studied for their antitumor effects in this neoplasia, the multitarget potential of these drugs, as we demonstrated in silico, was still unknown. Furthermore, the affinity of the 11 nsBBs currently prescribed for other pathologies for these new potential therapeutic targets was unknown. However, the limitation of this research is that it requires further experimental studies to demonstrate what the in silico analysis shows.

## 5. Conclusions

In this study, we identified for the first time through BIPA that nsBBs have affinity for key receptors involved in the pathophysiology of BC, such as ERBB2 and NPYR, in addition to ADRB2, which has already been extensively studied in this context. Furthermore, we demonstrated that other nsBBs, including carvedilol, carazolol, and labetalol, exhibit greater affinity for ADRB2 than propranolol. This raises questions about their potential antitumor effects in BC. Currently, experimental evidence supports only the antitumor effects of propranolol and carvedilol in BC via ADRB2; therefore, further studies are required to validate our findings. Finally, it is clear that nsBBs are promising candidates for BC adjuvant therapy, as clinical experience has shown that they are accessible in terms of cost and that their benefits outweigh the risks of adverse effects.

## Figures and Tables

**Table 1:** In vitro IC50 values of propranolol and carvedilol across various BC cell lines.

Drug Name	IC50 (μM)	Cell Line	Reference
Propranolol	100	MCF-7	[23]
Propranolol	180 to 261.5	MDA-MB-231	[24]
Propranolol	9.1	4T1	[25]
Propranolol	7.91	MDA-MB-231	[25]
Propranolol	179.0	MDA-MB-231	[26]
Propranolol	157.6	MCF-7	[27]
Propranolol	50.0	MCF-7	[28]
Carvedilol	98.75	MCF-7	[29]
Carvedilol	118.8	MDA-MB-231	[29]
Carvedilol	35.04	MDA-MB-231	[30]

**Table 2:** Gene Ontology (GO) terms associated with biological processes relevant to BC.

GO Term	Description	Strength	FDR ^1^	Matching Proteins
GO:0031622	Positive regulation of fever generation	2.85	0.0039	IL-1β, TNFSF11
GO:0031649	Heat generation	2.85	0.0039	IL-1β, ADRB2
GO:0033084	Regulation of immature T-cell proliferation in thymus	2.58	0.0054	IL-1β, ADRB2
GO:0032308	Positive regulation of prostaglandin secretion	2.55	0.0059	IL-1β, TNFSF11
GO:0045453	Bone resorption	2.45	0.0025	CTSK, ADRB2, TNFSF11

^1^ False discovery rate.

**Table 3:** Enriched KEGG pathways associated with the protein–protein interaction network relevant to BC.

KEGG Pathway	Description	Strength	FDR ^1^	Matching Proteins
hsa05144	Malaria	2.03	0.0180	IL-1β, SELP
hsa04923	Regulation of lipolysis in adipocytes	1.96	0.0180	ADRB2, NPY
hsa05323	Rheumatoid arthritis	1.95	0.0015	IL-1β, CTSK, TNFSF11
hsa04380	Osteoclast differentiation	1.79	0.0022	IL-1β, CTSK, TNFSF11
hsa04933	AGE-RAGE signaling pathway in diabetic complications	1.71	0.0376	IL-1β, VEGFC
hsa04064	NF-κB signaling pathway	1.69	0.0376	IL-1β, TNFSF11
hsa04620	Toll-like receptor signaling pathway	1.69	0.0376	IL-1β, CTSK
hsa04668	TNF signaling pathway	1.65	0.0376	IL-1β, VEGFC
hsa04010	MAPK signaling pathway	1.41	0.0180	IL-1β, ERBB2, VEGFC

^1^ False discovery rate.

**Table 4:** Docking results for BBs on ADRB2 (PDB: 6PS5), binding residues for each ligand and interactions.

Compound	Binding Energy (kcal/mol)	Interacting Residues		
		Hydrogen Bonds	Hydrophobic Interactions	Pi Interactions
**Stress Ligands**				
Epinephrine	−6.3	Asp113, Ser203	Val114, Phe290	Phe290
Norepinephrine	−6.3	Ser203, Asp113, Asn312	Phe290, Val114	Phe290
**Non-Selective Beta-Blockers**				
Propranolol	−8.5	Asp113, Asn312	His93, Phe193, Ile309	-
Carvedilol	−10.5	Asp113, Asn312, Tyr316, Ser203	Phe193, Trp109, Val114, Phe290, Ser207, Val117	-
Carazolol	−9.5	Asp113, Asn312, Ser203	Val114, Ser207, Phe290	-
Labetalol	−9.2	Asp113, Ser203,	Phe289, Phe290, Phe193, Trp109	Phe290
Timolol	−8.0	Asp113, Asn312	Phe193	Phe290
Pindolol	−7.9	Asp113, Asn312, Ser203	Val114, Phe290	-
Bupranolol	−7.6	Asp113, Asn312	-	-
Sotalol	−7.5	Asp113, Ser203, Ser204, Asn312	Phe193	-
Alprenolol	−7.0	Ser203, Ser204	Val117, Phe193	Phe289
Oxprenolol	−7.0	Asp113, Asn312	Trp109, Phe193	-
Nadolol	−6.5	Thr68, Asn69	Glu268, Ala271	-

**Table 5:** Docking results for nsBBs on ERBB2 (PDB: 3RCD), showing binding residues and interaction types.

Compound	Binding Energy (kcal/mol)	Interacting Residues		
		Hydrogen Bonds	Hydrophobic Interactions	Pi Interactions
Propranolol	−6.9	Asp863	Thr798, Leu785, Leu796, Lys753	-
Carvedilol	−9.1	Leu796	Leu852, Leu800, Phe864, Leu785, Lys753, Leu796	-
Labetalol	−8.8	Ala751, Ser783, Thr862	Leu852, Leu800, Val734, Ala751, Lys753, Leu785	-
Carazolol	−7.9	Leu796	Phe864, Leu785, Lys753, Leu796	-
Nadolol	−7.6	Thr862, Thr798, Ser783	Val734, Leu796, Lys753	-
Pindolol	−6.9	Thr862, Ala751	Lys753, Leu785, Thr798	-
Bupranolol	−6.9	Thr862	Thr798, Leu852	-
Sotalol	−6.7	Asp863, Thr862, Ser783, Thr798	-	-
Timolol	−6.4	Met801, Thr862	Leu800, Leu852	-
Alprenolol	−6.3	Gly865, Asp863	Lys753, Leu796, Leu785	-
Oxprenolol	−6.3	Asp863, Thr862	Ala751, Leu796, Leu785, Thr798, Lys753	-

**Table 6:** Docking results for nsBBs on the NPY receptor (PDB: 5ZBQ), showing binding energies and residue interactions.

Compound	Binding Energy (kcal/mol)	Interacting Residues		
		Hydrogen Bonds	Hydrophobic Interactions	Pi Interactions
Propranolol	−7.9	Asp104, Asn299	Ala294, Phe199, Asp104, Phe28	Phe28
Carvedilol	−9.2	Asn283, Asn299	Leu26, Phe173, Phe282, Phe286, Phe302	Phe173, Phe302
Nadolol	−9.0	Asn30, Ala294, Asn299	Phe28, Phe282	Phe28
Labetalol	−8.5	Asn299	Leu26, Phe199, Phe282 Ala294, Phe302	Phe199, Phe282, Phe286
Carazolol	−8.4	Thr295, Asn299, Phe302	Phe282, Ala294, Tyr100, Phe302	Phe302
Pindolol	−7.5	Asn299	Phe28, Asp104, Ala294	Phe28
Alprenolol	−7.5	Thr295, Asn299	Phe28, Asp104	Phe28
Bupranolol	−7.2	Asn299	Leu26, Phe28, Val197	-
Sotalol	−7.1	Asp283, His298, Asn299	Leu26, Phe282	Phe282, Phe286
Timolol	−7.0	Thr280, Asn283, Thr284	Phe173	-
Oxprenolol	−6.9	Asp104, Asn299	Phe302	Phe302

**Table 7:** RMSD values comparing the co-crystallized ligand pose in the PDB with redocking results using Vina and AutoDock4.

	Ligand	Vina	AutoDock4
ADRB2 (PDB: 6PS5)	Propranolol	0.48	2.00
ERBB2 (PDB: 3RCD)	03P *^1^	1.00	0.14
NPYR (PDB: 5ZBQ)	9AO *^2^	2.85	2.52

*^1^: PubCHEM ID:11620908, *^2^: PubCHEM ID: 127041619.

**Table 8:** RMSD for comparison between Vina pose with those from AutoDock4.

Compound	RMSD		
	ADRB2	ERBB2	NPYR
Propranolol	3.52	3.53	2.52
Carvedilol	3.36	4.22	2.42
Labetalol	1.77	2.36	2.39
Carazolol	0.83	1.91	2.24

## Data Availability

The original contributions presented in this study are included in the article/supplementary material. Further inquiries can be directed to the corresponding author.

## Supplementary Materials

The following supporting information can be downloaded at: https://www.mdpi.com/article/10.3390/cimb47100789/s1. Suplement 1. RMSD Images.

## Abbreviations

The following abbreviations are used in this manuscript:

ADRB2	beta-2-adrenergic receptor
ERBB2	v-erb-b2 avian erythroblastic leukemia viral oncogene homolog 2 (also HER2)
NPYR	Neuropeptide Y receptor Y1
PDB	Protein Data Bank
BC	Breast Cancer
nsBBs	Non selective betablockers
