# Supplementary material for: In Silico Multitarget Profiling of Non-Selective Beta-Blockers Highlights Their Potential as Key Agents in Breast Cancer Adjuvant Therapy via ADRB2, ERBB2, and NPYR Receptors
Source: Curr Issues Mol Biol. 2025 Sep 23;47(10):789. doi: 10.3390/cimb47100789 (PMC12564330; doi:10.3390/cimb47100789)
Supplement: Supplementary file 1 [file cimb-47-00789-s001.zip › Suplement 1. RMSD Images.pdf]

ADRB2 **(PDB: 6PS5) redocking**

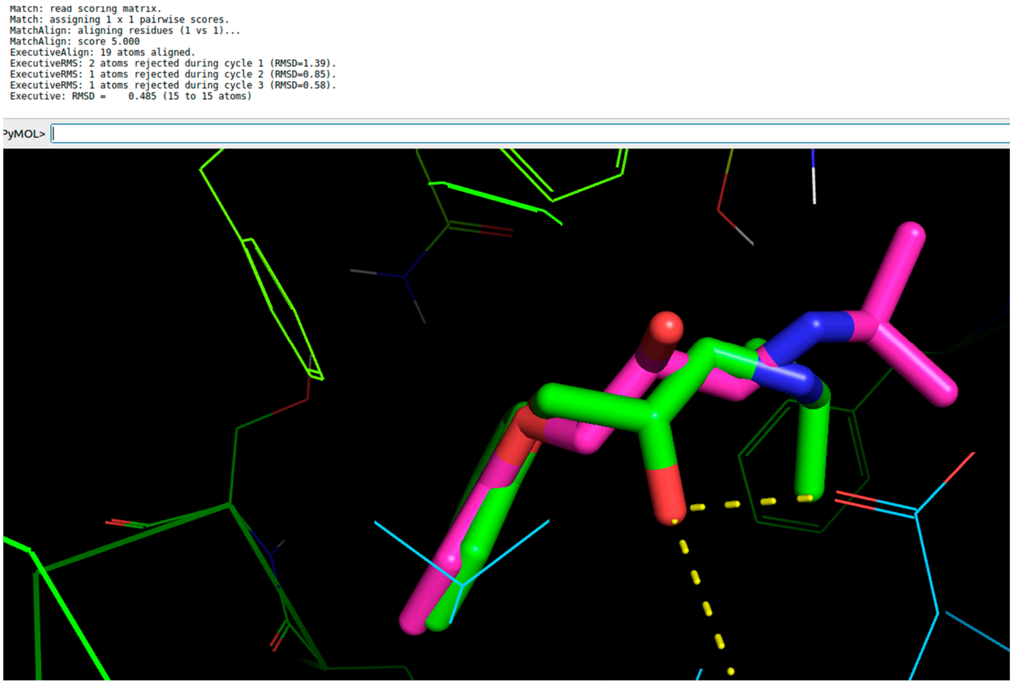

Figure S1. RMSD: Ligand Propranolol vs Vina Pose

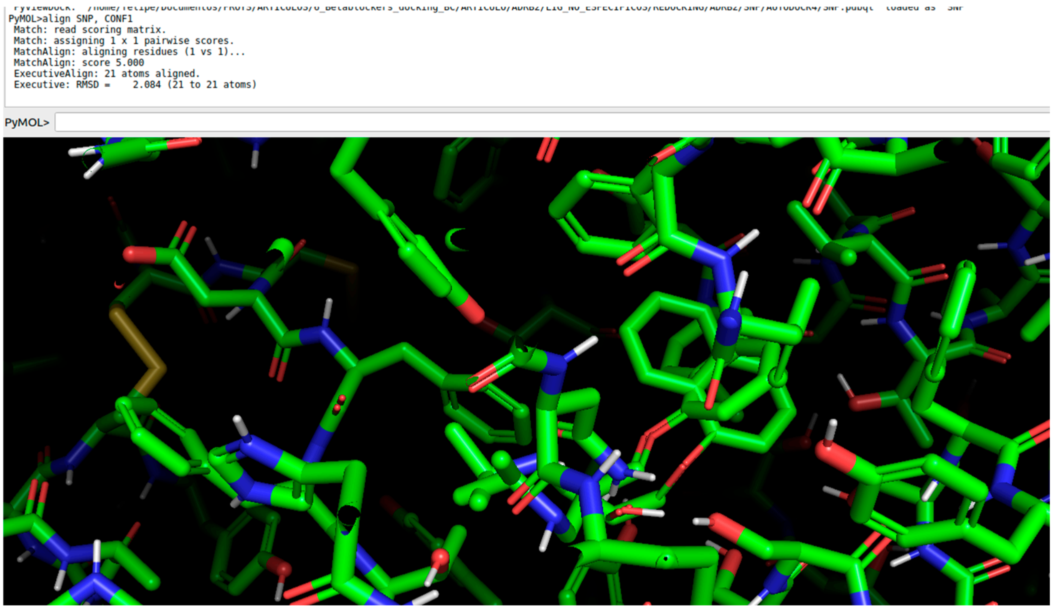

Figure S2: RMSD: Ligand Propranolol vs Autodock4 Pose

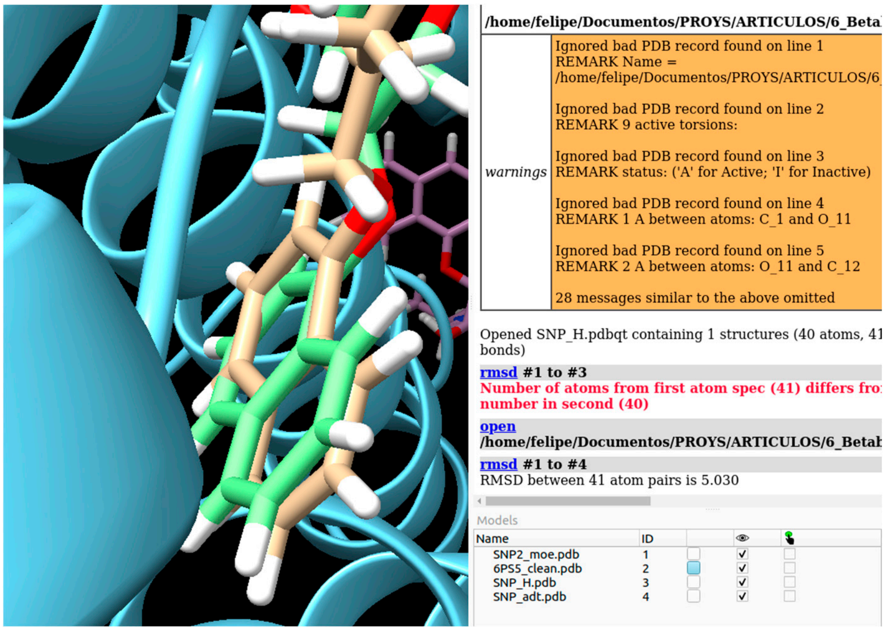

Figure S3: RMSD: Ligand Propranolol vs MOE Pose

ERBB2 **(PDB: 3RCD) redocking**

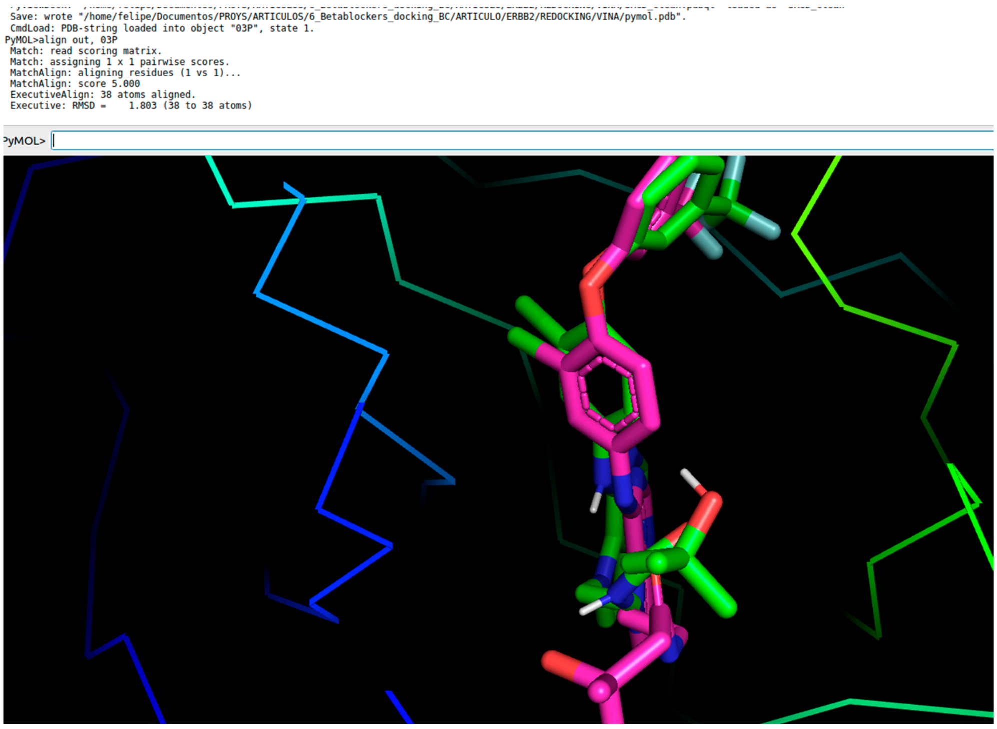

Figure S4. RMSD: Ligand 03P vs Vina Pose

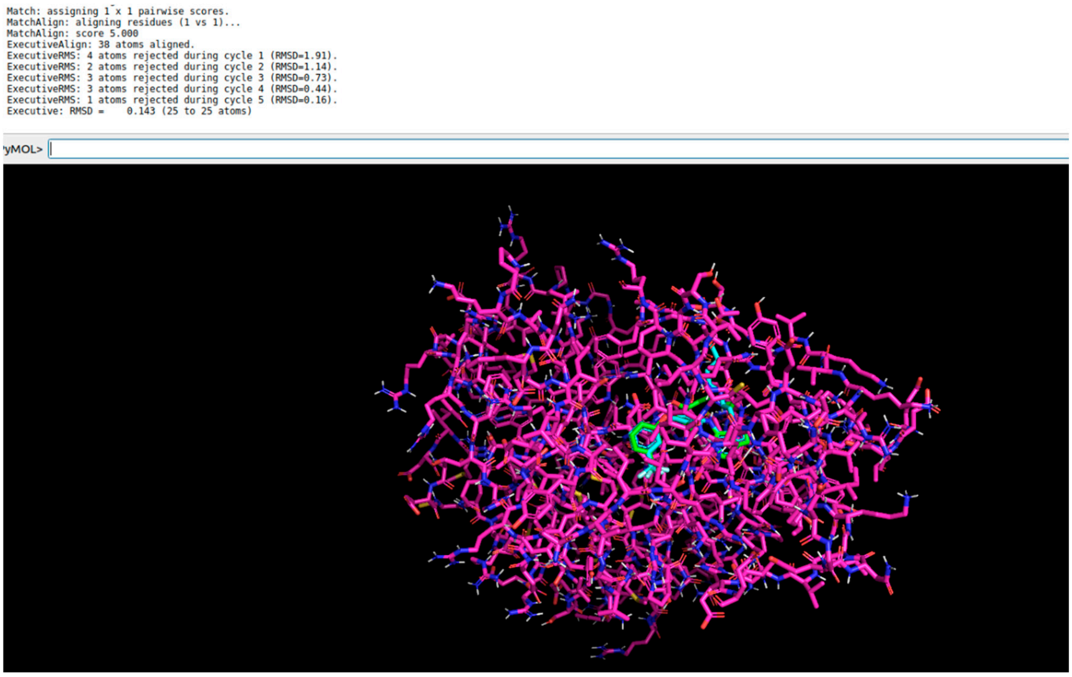

Figure S5: RMSD: Ligand 03P vs Autodock4 Pose

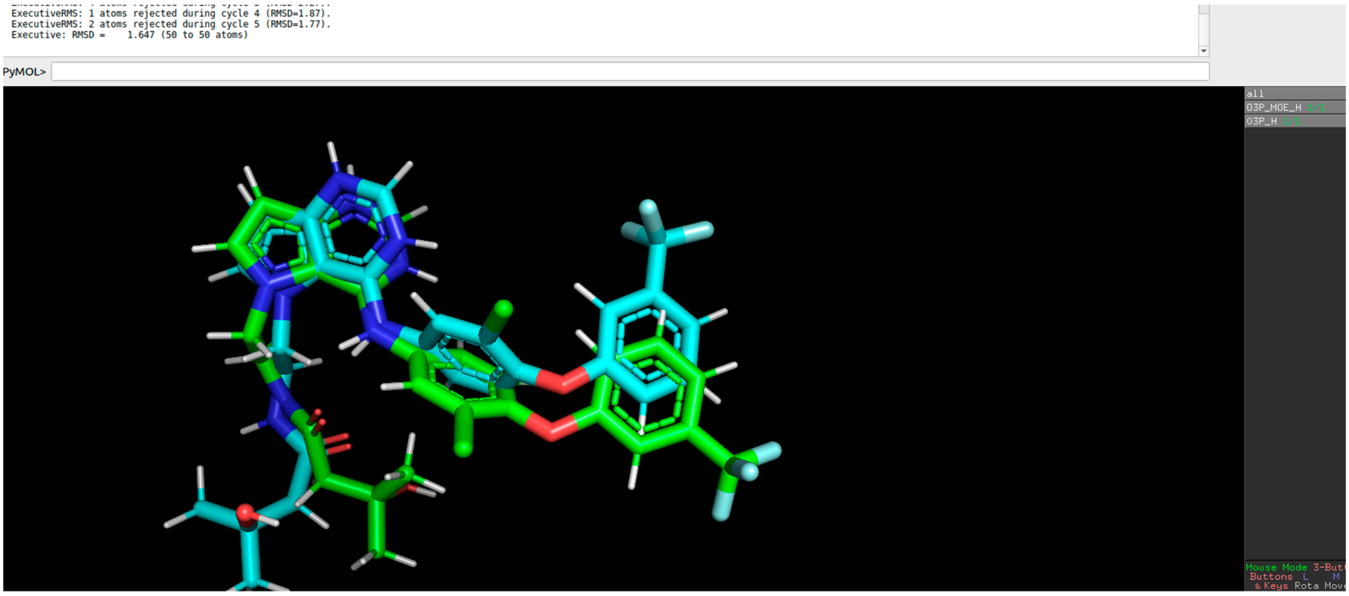

Figure S6: RMSD: Ligand 03P vs MOE Pose

NPYR (PDB: 5ZBQ) Redocking

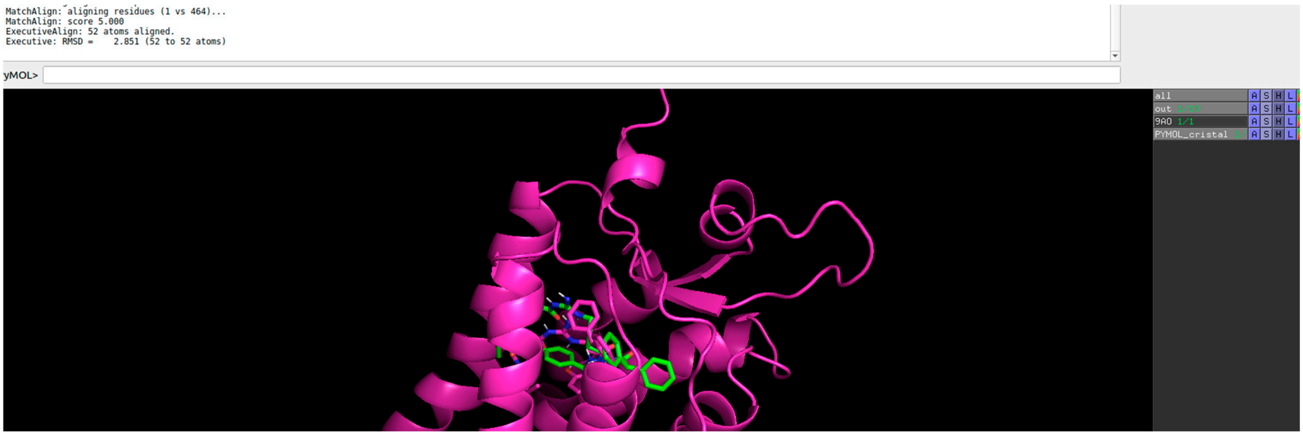

Figure S7: RMSD: Ligand 9AO vs VINA Pose

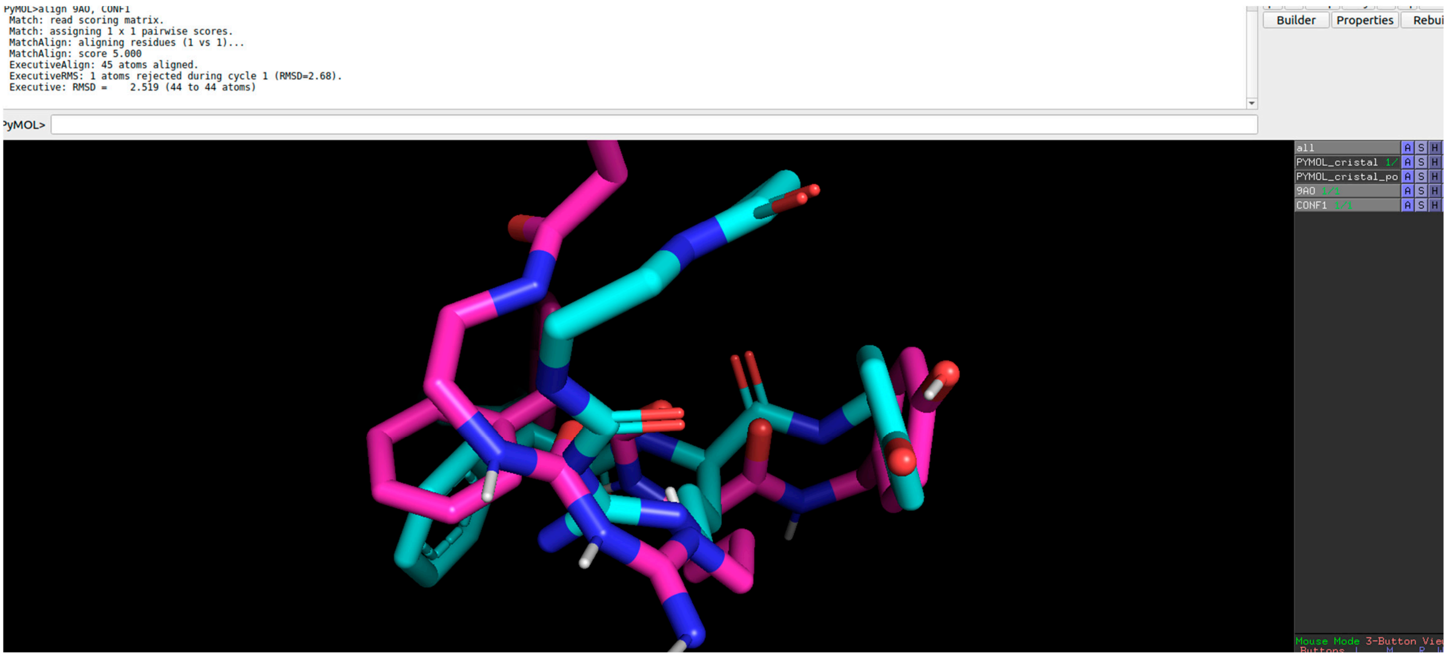

Figure S8: RMSD: Ligand 9AO vs Autodock4 Pose

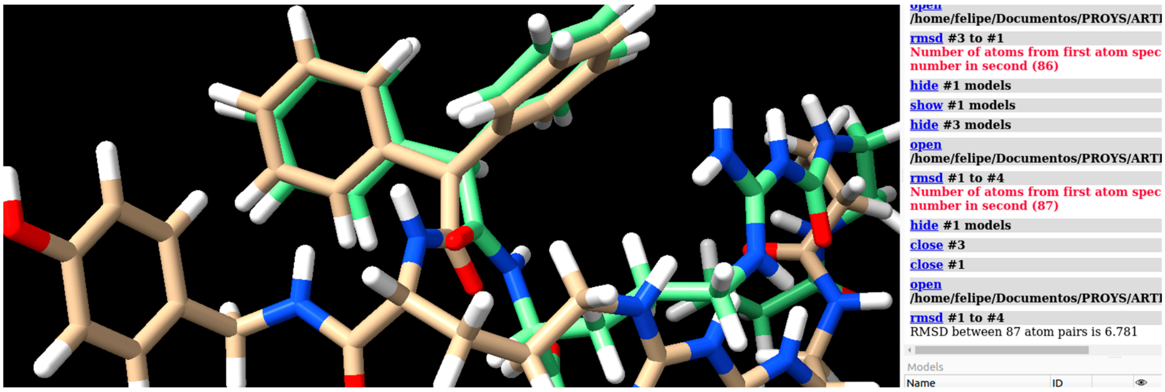

Figure S9: RMSD: Ligand 9AO vs MOE Pose
